# Nesidioblastosis with delayed diagnosis and post-operative complications in a patient with complex psychiatric history

**DOI:** 10.1192/bjo.2023.506

**Published:** 2023-10-19

**Authors:** Lee Ann Santore, Soumya Mandava, Gloria Lee, Mason Chacko

**Affiliations:** Renaissance School of Medicine, Stony Brook University Hospital, Stony Brook, New York, USA; Department of Psychiatry and Behavioral Health, Stony Brook University Hospital, Stony Brook, New York, USA

**Keywords:** Comorbidity, history of psychiatry, stigma and discrimination, borderline personality disorder, anorexia nervosa

## Abstract

Nesidioblastosis is a rare condition of organic persistent hyperinsulinaemic hypoglycaemia, with fewer than 100 cases since it was first recorded. However, an increasing prevalence suggests previous underdiagnosis due to poor knowledge and awareness. This case describes the presentation, clinical decision-making and unique challenges in diagnosis and care of a 21-year-old female with nesidioblastosis and extensive psychiatric comorbidities. She was repeatedly misdiagnosed until 2021, despite having presented to emergency departments with hypoglycaemic symptoms for over 7 years. Her symptoms were often misattributed to behaviours secondary to restrictive anorexia nervosa and borderline personality disorder. Even after appropriate diagnosis and management, she suffered a complicated post-operative course. Patients with psychiatric comorbidities are at higher risk of distress, communication difficulties and inadequate social support, all of which could be better managed with increased multidisciplinary collaboration between endocrine, surgery, psychiatry, pain management and social work. This study highlights the importance of well-rounded patient care that addresses all facets of patient health. This approach not only improves quality of care, but also reduces overall readmissions, revisions, morbidity and mortality.

Nesidioblastosis is a rare condition of organic persistent hyperinsulinaemic hypoglycaemia. Proposed mechanisms include pathological overgrowth from pancreatic ductal epithelial cells differentiating and forming beta islet cell hyperplasia, or dysregulation of function of beta islet cells. It occurs in newborns and, more rarely, in adults. Since the first recorded case of adult-onset nesidioblastosis in 1975, fewer than 100 cases have been reported. The relative frequency has been estimated to be 0.5–0.7%, with a slowly increasing prevalence, suggesting that nesidioblastosis may have been previously underdiagnosed owing to poor knowledge and awareness of the condition.^1^ The presentation is nearly identical to that of an insulinoma, which is the most common cause of adult-onset primary hyperinsulinaemic hypoglycaemia. The clinical manifestations include diaphoresis, tremors, nausea, vomiting, headache and, in more severe cases, loss of consciousness, confusion and seizures. The vague nature of these symptoms results in significant misdiagnosis and delayed diagnosis.^2^ The prolonged course of hypoglycaemia in these patients, prior to their diagnosis, can have long-term damaging effects on their neuropsychiatric state. The effects of the hypoglycaemia may be reversible secondary to treatment. The most effective treatment modality for nesidioblastosis is surgical resection of the pancreas, ranging from distal pancreatectomy to total pancreatectomy.^1^

The diagnosis can be further complicated by the presence of neuropsychiatric comorbidities. Although there is a gap in the literature regarding nesidioblastosis specifically, a detailed review was carried out for 42 patients with insulinoma, which presents clinically identical to nesidioblastosis. Of these 42 insulinoma patients, 25 had neuropsychiatric symptoms. Of the 17 patients without neuropsychiatric symptoms, 64.7% were diagnosed within 1 month of seeking consultation and none had received a prior misdiagnosis. Of the patients with neuropsychiatric symptoms, 64% did not receive an accurate diagnosis until more than 1 year after initial symptom presentation. Before being appropriately diagnosed, 100% of the patients with neuropsychiatric manifestations were misdiagnosed at least once, with conditions such as epilepsy, transient ischaemic attack, stroke, hysteria and narcolepsy. Symptoms that confounded initial appropriate diagnosis include confusion, memory disorder, abnormal behaviour, hysteria, personality change, weakness and convulsions. Notably, even imaging studies such as ultrasound, computed tomography (CT), magnetic resonance imaging (MRI) and digital subtraction angiography showed reduced sensitivity of morphological investigations in patients with neuropsychiatric symptoms compared with those without.^3^

We present here a case of nesidioblastosis in a patient with multiple psychiatric comorbidities, including anorexia nervosa, borderline personality disorder, anxiety and a history of addiction. The patient's extensive neuropsychiatric presentations likely exacerbated her delayed diagnostic course and led to multiple post-surgical care complications after pancreatectomy.

## Patient consent and ethical practices

Verbal informed consent was obtained from the patient prior to developing this case report. Owing to the nature of the case report, no approval was required from an ethics review body.

## Presentation

A 22-year-old female was brought by ambulance to our emergency department in August 2021 for diaphoresis and weakness. She was found to have hypoglycaemia with fingerstick glucose in the 20s, refractory to glucagon. She attempted to leave against medical advice. However, psychiatry deemed her not to have capacity, and she was admitted to the medical intensive care unit.

### Medical and psychiatric history

She had a medical history of nesidioblastosis status after a distal pancreatectomy 8 months earlier. Her psychiatric history included diagnoses of anxiety, depression, attention-deficit hyperactivity disorder, obsessive–compulsive disorder, cluster B traits, specifically borderline personality disorder, substance use disorder requiring in-patient detoxification and anorexia nervosa requiring treatment at an eating disorder clinic. In August of 2021, her psychiatric conditions were managed with dialectic behavioural therapy (DBT), psychotherapy, sertraline, aripiprazole, methamphetamine and lorazepam. As per patient report, she was intermittently non-adherent to her psychiatric medications owing to concerns regarding her weight gain.

She had frequent visits to the emergency department for hypoglycaemia over the past 7 years. These visits required psychiatry consultations to ascertain capacity, as she often attempted to leave against medical advice. She began developing hypoglycaemic seizures in October 2020, and was subsequently evaluated by surgical oncology in November 2020 to rule out insulinoma. Neither CT nor MRI revealed lesions of the pancreas. Hepatic venous sampling performed in December 2020 localised hyperinsulinaemia to the superior mesenteric and splenic arteries, prompting a planned distal pancreatectomy in January 2021. Pathology of the removed pancreatic tail did not reveal localised lesions; the findings were pathognomonic for nesidioblastosis. During the following few months, the patient did not have any complications or adverse effects from the procedure, but then had three episodes of hypoglycaemia requiring admission to a medical intensive care unit between May and August 2021. The decision was made to perform a pylorus-sparing robotic pancreaticoduodenectomy with excision of the remaining pancreas and reconstruction with choledochojejunostomy and gastrojejunostomy in September 2021. The patient consented to the procedure because she felt that iatrogenic type 1 diabetes mellitus would be more manageable than repeated hypoglycaemic events.

### Post-operative hospital course

One week after the pancreaticoduodenectomy, the patient reported frequent issues with pain control and sleep. She underwent several failed trial discontinuations of a hydromorphone patient-controlled analgesia (PCA) pump on post-operative days (POD) 4 to 8, resulting in the patient threatening to leave against medical advice if the pump was not restarted. Psychiatry was consulted to evaluate for capacity to leave against medical advice, particularly in the setting of new-onset insulin-dependent diabetes. It was determined that the patient did have capacity to leave, but she vomited on the way out and decided to stay. She was found to have post-operative delayed gastric emptying, and a nasogastric tube was placed. On POD9, the patient removed the nasogastric tube herself because she felt it was a traumatising experience having the tube inserted and in place. She remained nothing by mouth because of nausea and vomiting from that point onwards. On the morning of POD12, she began screaming and acting out against staff owing to pain. During this episode she expressed a wish to die because of the pain. As a result, the patient was placed on a one-to-one watch and received haloperidol. Later that day, when psychiatry came to evaluate her, she said that she had no wish to die and denied suicidal or homicidal thoughts. She also said that she would not consent to a nasogastric tube.

Despite extensive counselling from surgical and psychiatric teams, she continued to refuse a nasogastric tube. On POD13, a peripherally inserted central catheter (PICC line) was placed and she was started on total parenteral nutrition. On POD16, a percutaneous endoscopic gastrostomy (PEG) tube was placed.

### Long-term follow-up

The patient continues to experience abdominal pain and bouts of nausea and vomiting. She has undergone a diagnostic endoscopy and had her PEG tube removed and replaced. She has had several episodes of both hyperglycaemia and hypoglycaemia secondary to both dietary and insulin non-adherence. She has made several attempts to self-harm.

## Discussion

This patient had a prolonged diagnostic and treatment course that may in part be attributed to her non-specific clinical presentation and the presence of neuropsychiatric manifestations. Her post-operative course was further complicated by her psychiatric comorbidities.

### Variation in presentation of nesidioblastosis

One of the challenges of diagnosing nesidioblastosis is the variation in patient presentation. Less severe symptoms, such as diaphoresis, chills, night sweats, headaches, dizziness, lightheadedness, lethargy and anxiety, may be overlooked without healthcare follow-up because patients may be unconcerned by their occurrences. As the hypoglycaemic state persists, however, the symptoms become more severe. Reports have shown primary complaints including seizures, weight gain greater than 30 kg, palpitations, confusion, forgetfulness, dysphagia, dysarthria and diplopia. None of these symptoms, however, are pathognomonic to persistent hyperinsulinaemic hypoglycaemia. Owing to the relatively low prevalence of nesidioblastosis, healthcare workers may miss diagnostic consideration of this condition in patients presenting with these non-specific symptoms. Studies report most patients having months to years between their initial presentation of symptoms to a physician and their final diagnosis of nesidioblastosis.^4^ In the present case, the patient's symptoms included anxiety, lethargy, diaphoresis, weakness and seizures. Her hypoglycaemia in past presentations to the emergency department was often misattributed to behaviours secondary to restrictive anorexia nervosa and borderline personality disorder. She was not appropriately diagnosed with nesidioblastosis until 2020, despite having presented to emergency departments with hypoglycaemic symptoms for over 7 years.

### Psychiatric comorbidities complicate post-operative course of patient

Multiple studies have shown a negative relationship between pre-existing psychiatric conditions and post-operative recovery. One study compared the number of days spent in the hospital between patients with and those without comorbid psychiatric disorders, finding that those with comorbid psychiatric disorders stayed an average of 28.63 days, whereas those without psychiatric comorbidities stayed only 13.78 days. Similarly, there was a statistically significant increase in the number of procedures the patients with psychiatric comorbidities required.^5^ Another study reported on 412 777 elective orthopaedic patients, who were grouped based on the presence of psychiatric diagnoses (‘psychiatric’) or the lack thereof (‘non-psychiatric’). In this study, the psychiatric diagnoses that were included were adjustment disorders, anxiety disorders, cognitive dysfunction, mood disorders, personality disorders, substance use disorders and history of suicidal ideation or self-harm. When comparing the post-operative complication rates of these patients, 30.2% suffered complications in the psychiatric group, compared with 25.1% in the non-psychiatric group. The mean length of stay was 4.36 days for the psychiatric group and 4.25 days for the non-psychiatric. Finally, the risk of mortality in the psychiatric group was 1.43, compared with 1.40 in the non-psychiatric.^6^ It was hypothesised that the reason for these differences is that psychiatric patients have overall greater psychological distress, impaired cognition, poor appetite, low mood, poor motivation, communication difficulties, adherence and behavioural issues, and inadequate social support at home. These factors may compromise their recovery and lead to delays in seeking healthcare, which increase their risk of infection, delayed wound healing, delayed functional recovery and increased complication rates, thus requiring further procedures and therapeutics.^5^

In the present case, the patient had significant psychiatric manifestations that affected her post-operative care. Her history of addiction made it difficult to wean her off PCA and prolonged her hospital stay. Owing to her borderline personality disorder diagnosis and consequent splitting, the management teams had to work carefully to ensure that she understood the proposed care plans and felt that they were in her best interest and that the team remained on the ‘good side’ of her split to encourage adherence, communication and follow-up. Her anxiety disorder worsened her fear regarding emesis and delayed restarting her oral intake. Last, her eating disorder had caused her previously to stop taking her medications owing to concern of weight gain. Since the patient was aware that weight gain is a potential side-effect of insulin, it was important that the team appropriately manage her anorexia, so she would not discontinue her prescribed insulin despite her new diagnosis of insulin-dependent diabetes. Throughout her in-patient course, the psychiatry team was involved and helped alleviate some of the care concerns attributed to her comorbidities by navigating challenging conversations and decision-making discussions with her and her family. A notable area of improvement for future patients would be implementation of psychotherapy. In this case, it might have been beneficial to incorporate routine DBT sessions, which she has responded to previously, as their use during her admission could have provided extra support. Additionally, the psychiatry team could have been involved in overseeing and encouraging multidisciplinary coordination with social work, endocrine, surgery and pain management to provide further comprehensive, well-rounded care that addressed all her needs.

## Conclusion

The present case stresses the importance of testing for endogenous or exogenous insulin effects when patients present with new-onset vague neuropsychiatric manifestations. The effects of chronic or recurrent long-standing hypoglycaemia can be debilitating and irreversible, despite being easily preventable. Therefore, it is prudent to include a fingerstick glucose test as part of acute psychiatric presentation workups. Additionally, it provides another example of how psychiatric care is a vital aspect of patient care, even when the hospital admission is not for psychiatric reasons. Patient care that addresses all facets of the patient's health not only improves quality of care, but also leads to overall decreases in readmissions, revisions, morbidity and mortality in this population.

## Data Availability

Data availability is not applicable to this article as no new data were created or analysed in this study.
